# Wood’s Medical and Surgical Monographs

**Published:** 1889-08

**Authors:** 


**Wood's Medical and Surgical Monographs. Volume II., No. 3, June, 1889.** Contents: General Orthopedics, including Surgical Operations. By DR. AUGUST SCHREIBER. Index for Vol. II. Pp. xii., 336.

**Volume III., No. 1. July, 1889.** Contents: Cancer and Cancerous Diseases. By SIR SPENCER WELLS, Bart., F. R. C. S. Cardiac Dyspnea and Cardiac Asthma. By DR. S. VON BASCH. The Influence of Menstruation and of the Pathological Condition of the Uterus on Cutaneous Diseases. By DR. L. GRELLETY. Tension as met with in Surgical Practice; Inflammation of Bone; Cranial and Intercranial Injuries. By T. BRYANT, F. R. C. S. Antisepsis, and its Relation to Bacteriology. By DR. J. NEUDORFER. Pp. ii., 254. New York: Wood & Co. Published monthly. $10 a year. Single copies, $1.

I. Whoever has attentively observed the advances made in the surgical field during the past quarter of a century, cannot fail to be impressed with the fact that nowhere has this improvement been more apparent or substantial than in orthopedics. While it is not possible for the general practitioner to equip himself to deal with all the deformities that befall man, he should yet be sufficiently familiar with them in general to direct their management into proper channels, and to be able to supervise the intermediate details. In Dr. Schreiber's treatise will be found the latest thought on the subject. It is copiously illustrated with 388 handsome cuts, that serve to elucidate the text in an admirable manner. The index to Volume II. accompanies this number.

II. With the July number begins Volume III., and the several subjects therein contained deserve to be read carefully to be appreciated. Every one of the subjects are of sufficient importance to demand this, and no cursory review can do them justice. The standard of these monographs has thus far been maintained even beyond the promise of the publishers in their announcement. No error can be committed in the purchase of these books, singly or in groups.

